# Supplementary material for: EGFR-Binding Peptides: From Computational Design towards Tumor-Targeting of Adeno-Associated Virus Capsids
Source: Int J Mol Sci. 2020 Dec 15;21(24):9535. doi: 10.3390/ijms21249535 (PMC7765298; doi:10.3390/ijms21249535)
Supplement: Supplementary file 1 [file ijms-21-09535-s001.pdf]

Supplementary Material

EGFR-Binding Peptides: From Computational Design towards Tumor-Targeting of Adeno-Associated Virus Capsids

Rebecca C. Feiner [1,2], Isabell Kemker [3,4], Lea Krutzke [5], Ellen Allmendinger [5], Daniel J. Mandell [6,7,8], Norbert Sewald [3], Stefan Kochanek [5] and Kristian M. Müller [1,*]

1 Cellular and Molecular Biotechnology, Faculty of Technology, Bielefeld University, 33615 Bielefeld, Germany; rebecca.feiner@uni-bielefeld.de
2 Present address: Freeline Therapeutics, Stevenage Bioscience Catalyst, Gunnels Wood Road, Stevenage SG1 2FX, UK
3 Organic and Bioorganic Chemistry, Faculty of Chemistry, Bielefeld University, 33615 Bielefeld, Germany; isabell.kemker@uni-bielefeld.de (I.K.); norbert.sewald@uni-bielefeld.de (N.S.)
4 Present address: Roche Diagnostics, 82377 Penzberg, Germany
5 Department of Gene Therapy, Ulm University, 89081 Ulm, Germany; lea.krutzke@uni-ulm.de (L.K.); ellen.allmendinger@uni-ulm.de (E.A.); stefan.kochanek@uni-ulm.de (S.K.)
6 Department of Bioengineering and Therapeutic Sciences, University of California San Francisco, San Francisco, CA 94158, USA; dan.mandell@grobio.com
7 Bioinformatics Graduate Program, University of California San Francisco, San Francisco, CA 94134, USA
8 Present address: GRO Biosciences, 700 Main Street North, Cambridge, MA 02139, USA
* Correspondence: kristian@syntbio.net; Tel.: +49-521-106-6323

Table of Content

1 Supplementary Figures 2
2 Analytical Data for Cyclization 7
3 Analytical Data of Final Compounds 10
4 Western Blot Raw Images 16
5 High Resolution Mass Spectrometry 16
6 General Analytical Methods 16
7 Literature 17

1 Supplementary Figures

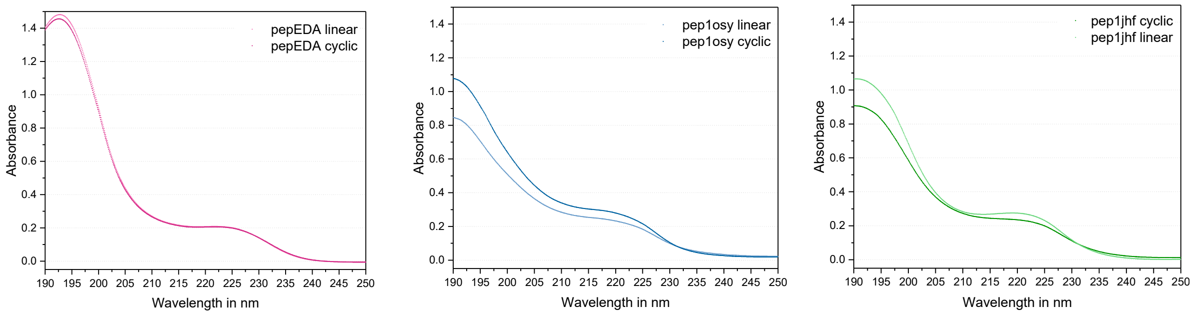

Figure S1. Absorption spectra for pepEDA, pep1osy and pep1jhf at 100 µM recorded during CD measurements in 10 % TFE/water (v/v) from 190 to 250 nm at 25 °C.

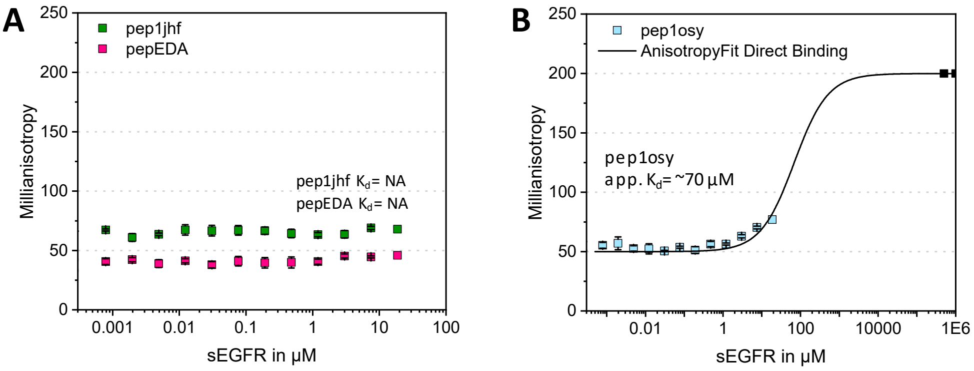

Figure S2: Fluorescence polarization assay for determination of the dissociation constant of the FAM-labeled peptides and soluble EGFR. Errors were calculated for three independent replicates using Gaussian error propagation. A) Anisotropy data for pep1jhf and pepEDA show no change in the anisotropy signal. Calculation of the dissociation constant could not be performed. B) For fitting a value of 0.2 anisotropy (added as black squares) was assumed for 1 M EGFR based on previous experiments with proteins and peptides of similar molecular weight and a 0.15 A change in anisotropy. This approximation results in an apparent K_d for pep1osy of 70 µM.

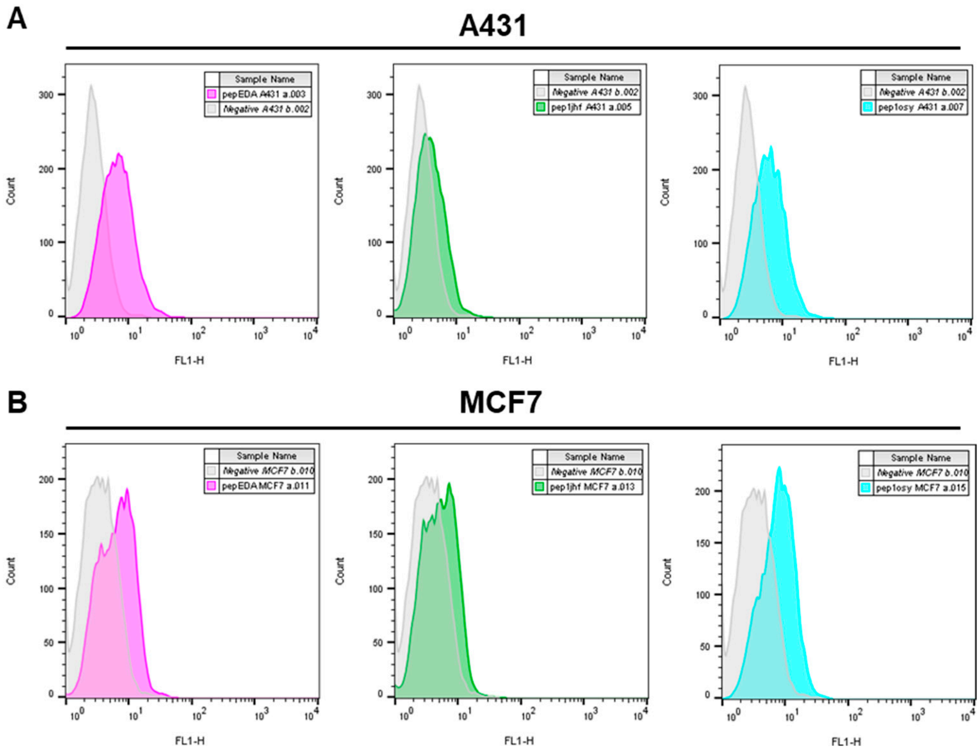

Figure S3. Flow cytometry data analysis of cyclic peptides on A431 (A) and MCF7 (B) cells. Cells were incubated with 20 μM peptide for 15 min at 37 °C before measuring fluorescence signal via flow cytometry using a FACScalibur system. Overlay histograms were created using FlowJo.

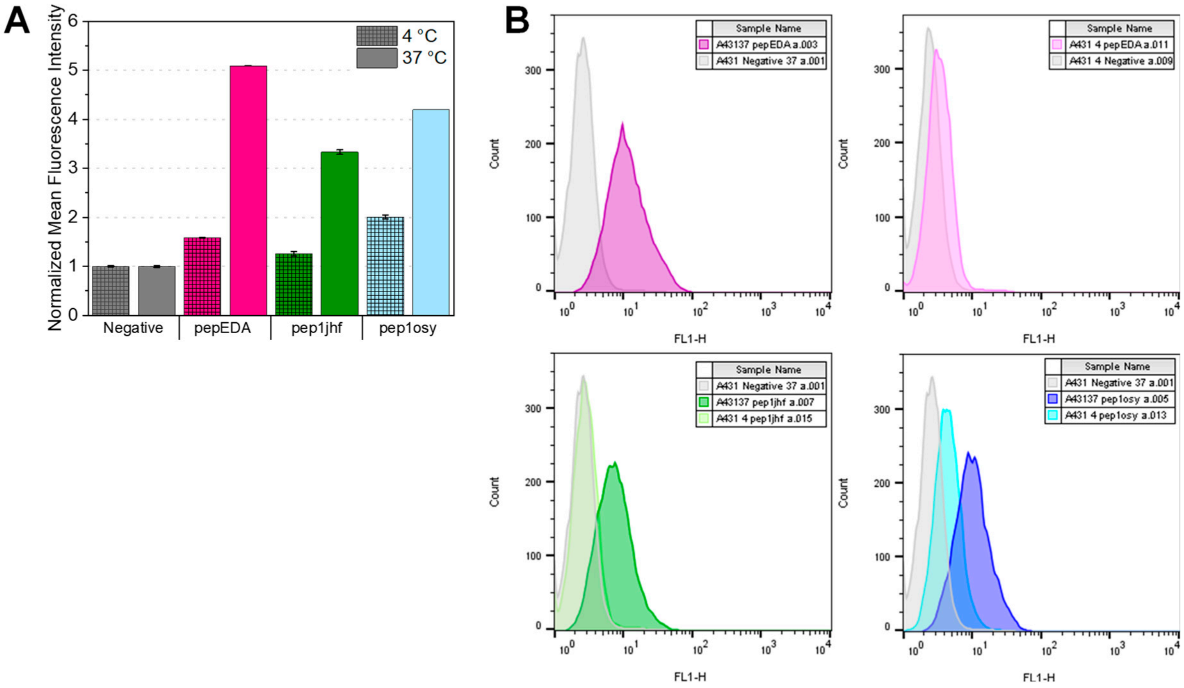

Figure S4. Flow cytometry analysis of cyclic peptides on A431 cells. (A) Normalized mean fluorescence intensity. (B) Overlay histograms of flow cytometry data. Cells were incubated with 20 μM peptide for 15 min at 4 °C and 37 °C before measuring fluorescence signal via flow cytometry using a FACScalibur system.

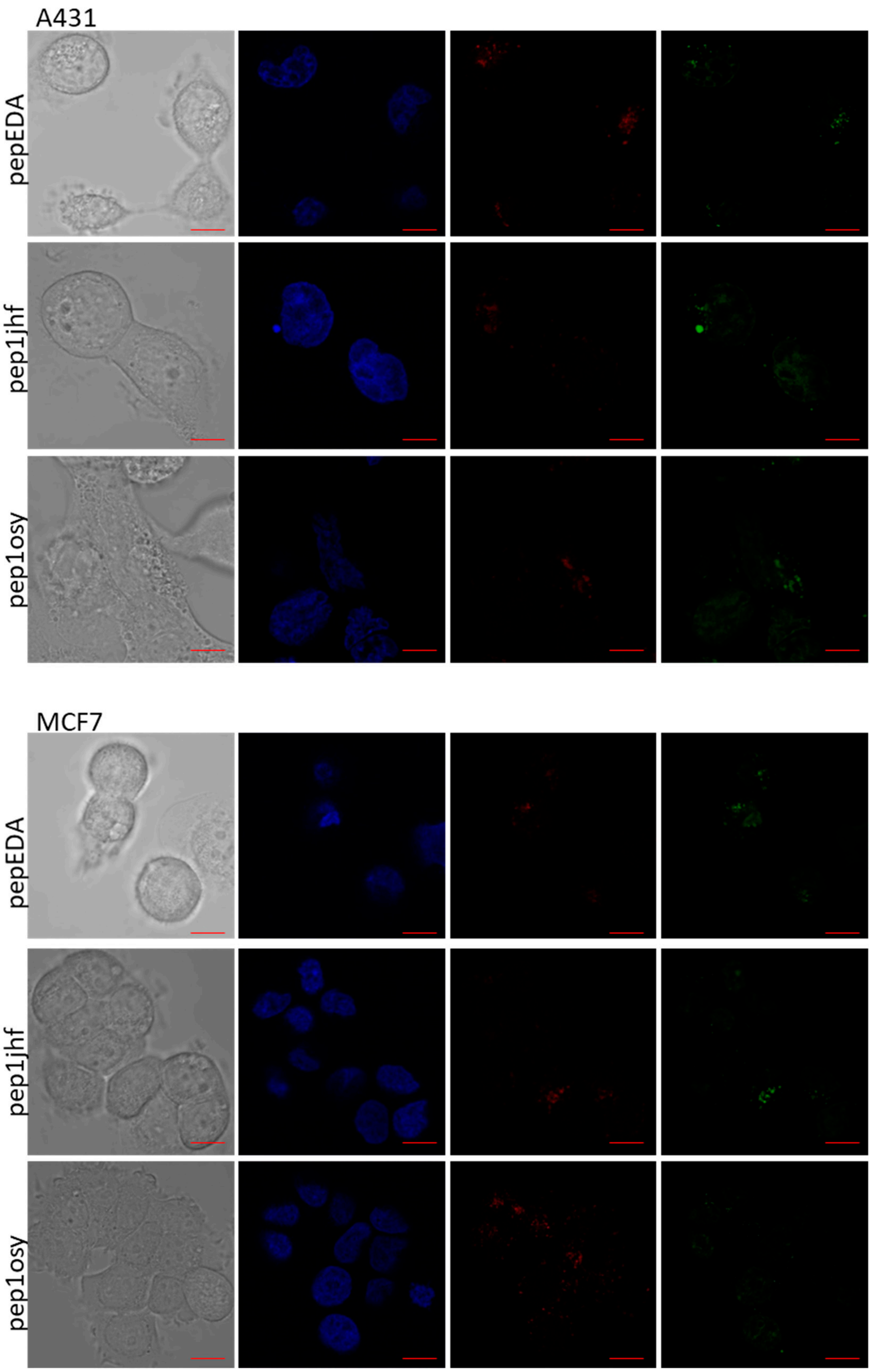

Figure S5. Live cell imaging of cyclic FAM-labeled peptide variants incubated with A431 and MCF7 cells for 10 min at 5 µM. Nuclei and lysosomes were counterstained using NucBlue (second row) and Lysotracker DND-99 (third row), respectively. Fluorescence microscopy was performed at 63× magnification using an inverted laser scanning Zeiss LSM780 microscope. Scale bars are highlighted in red and represent 10 µm.

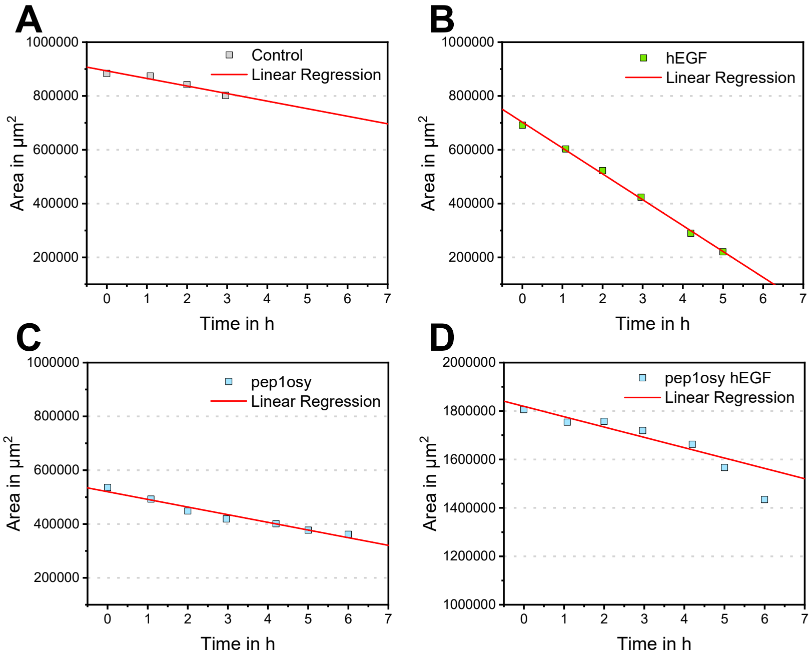

Figure S6. Wound healing assay analysis. The gap area over time is analyzed for each sample. Areas were calculated using ImageJ. Linear regressions were calculated to determine to growth rate. Exemplary the data analysis for control (A), hEGF (B), pep1osy (C) and pep1osy hEGF (D) are presented for one replicate.

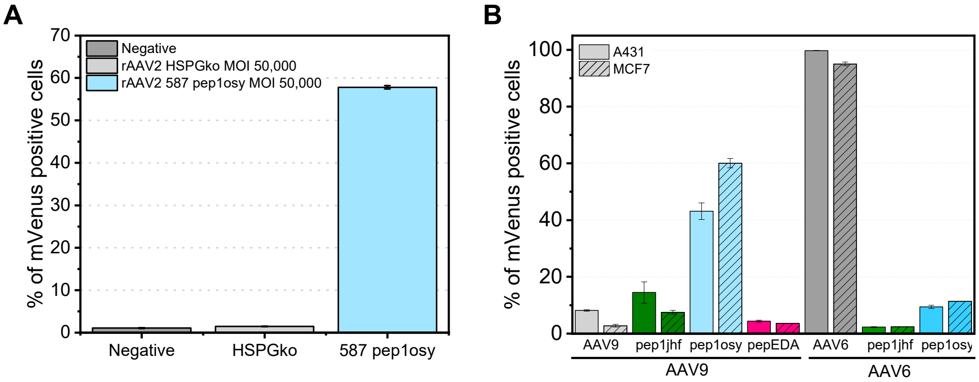

Figure S7. Transduction experiments. (A) Transduction experiments with an independent rAAV preparation to demonstrate the reproducibility of the experiments. Pep1osy-carrying AAV2 shows significantly enhanced transduction of EGFR-expressing cells in comparison to the rAAV2 ΔHSPG variant. A431 cells were transduced with mVenus-expressing rAAV peptide variants at a MOI of 50,000. mVenus expression levels of biological duplicates were analyzed 96 hours post transduction by flow cytometry. (B) A431 and MCF7 cells were transduced at an MOI of 50,000 and analyzed for mVenus expression 96 h post transduction. AAV6 and AAV9 vectors with the wild-type capsid are compared to the peptide modified variants. We chose especially AAV6 because it was shown to have a natural tropism towards the EGFR and we thought the incorporation of EGFR-specific peptide might even increase the transduction ability [1]. Plasmids coding for Cap6 and Cap9 proteins were modified to allow for the insertion of peptides in the same position that was previously analyzed for each serotype [2,3].

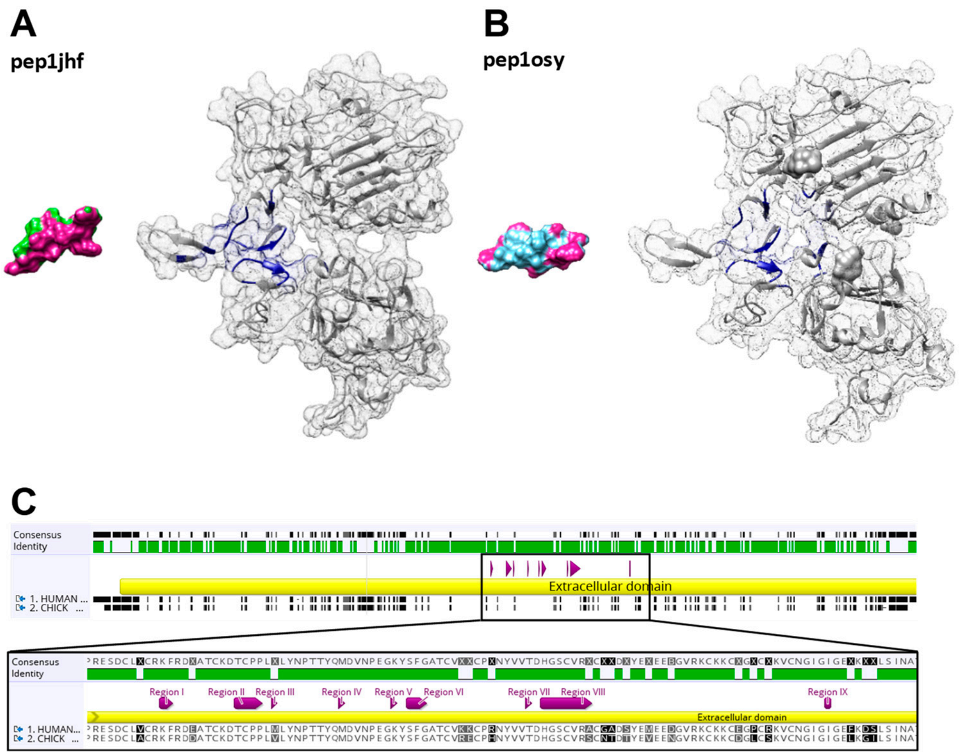

Figure S8. Identification of the binding interface of pep1jhf and pep1osy. PDB files of the bound peptide to the EGFR are the results of the rational peptide design of pep1jhf (A) and pep1osy (B). Contacts between atom pairs of peptide and receptor were determined using UCSF Chimera. A cut-off of 1.0 Å distance was defined to identify atoms pairs. The binding interface is highlighted in magenta (peptide) and dark blue (EGFR). (C) Alignment of the human EGFR (UniProt P00533) and the chicken EGFR (UniProt A0A1D5NZB4) was generated using Geneious. Regions identified from previous contact determination are highlighted in magenta.

2 Analytical Data for Cyclization

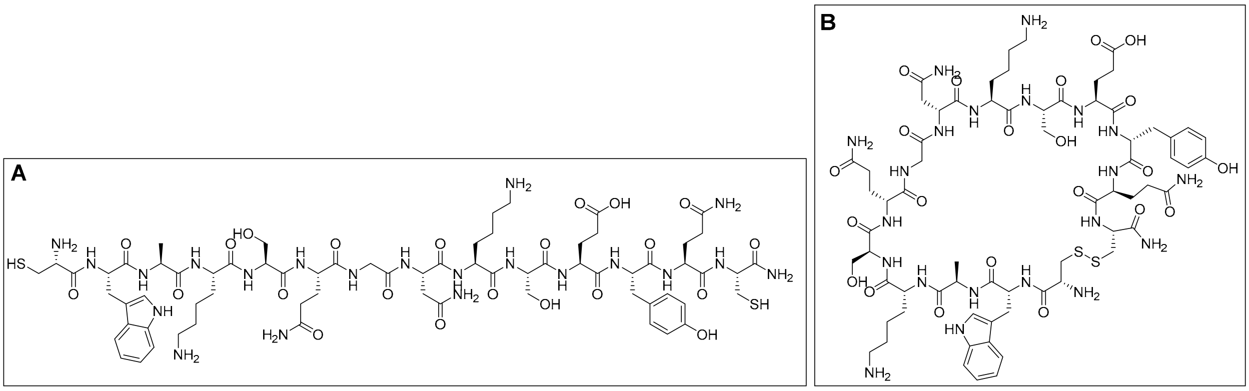

Cys-Trp-Ala-Lys-Ser-Gln-Gly-Asn-Lys-Ser-Glu-Tyr-Gln-Cys

Figure S9. Structure and sequence information for pep1jhf in linear (A) and cyclic (B) variant.

<u>**A** linear;</u> *m*/*z* calculated for $C_{68}H_{103}N_{21}O_{22}S_2$; $[M+2H]^{++}$ = 815.8; found = 815.8

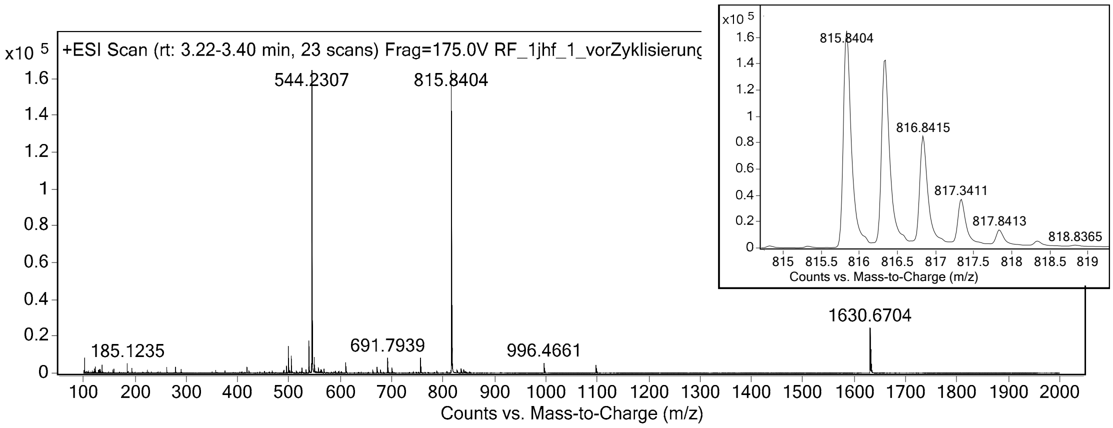

<u>**B** cyclic;</u> *m*/*z* calculated for $C_{68}H_{101}N_{21}O_{22}S_2$; $[M+2H]^{++}$ = 814.8; found = 814.8

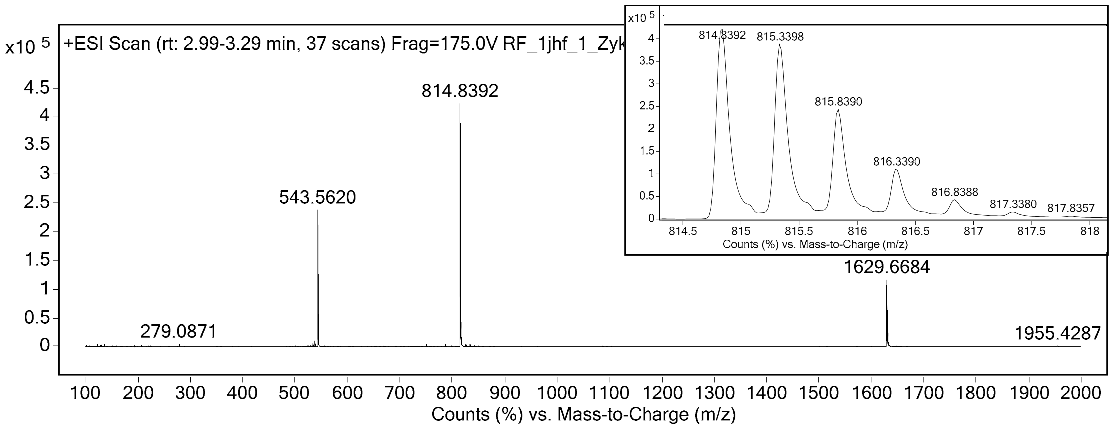

Figure S10. TIC spectra of LC-MS analysis of crude linear (A) and cyclic (B) peptide to verify cyclization by disulfide bridge formation.

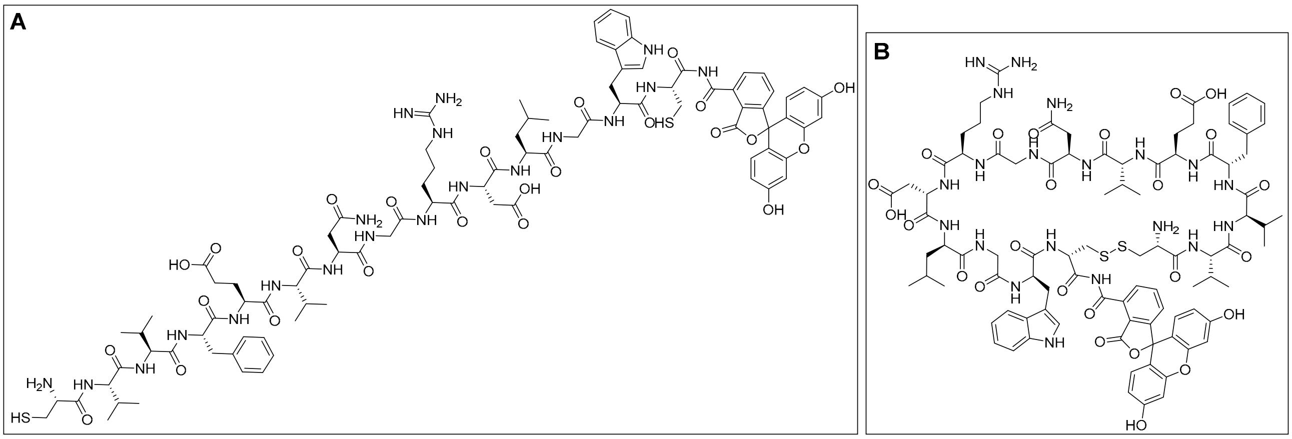

Cys-Val-Val-Phe-Glu-Val-Asn-Gly-Arg-Asp-Leu-Gly-Trp-Cys

Figure S11. Structure and sequence information for FAM-labeled pep1osy in linear (A) and cyclic (B) variant.

<u>**A** linear FAM-labeled;</u> *m/z* calculated for $C_{91}H_{116}N_{20}O_{25}S_2$; $[M+2H]^{++}$ = 977.4; found = 977.4

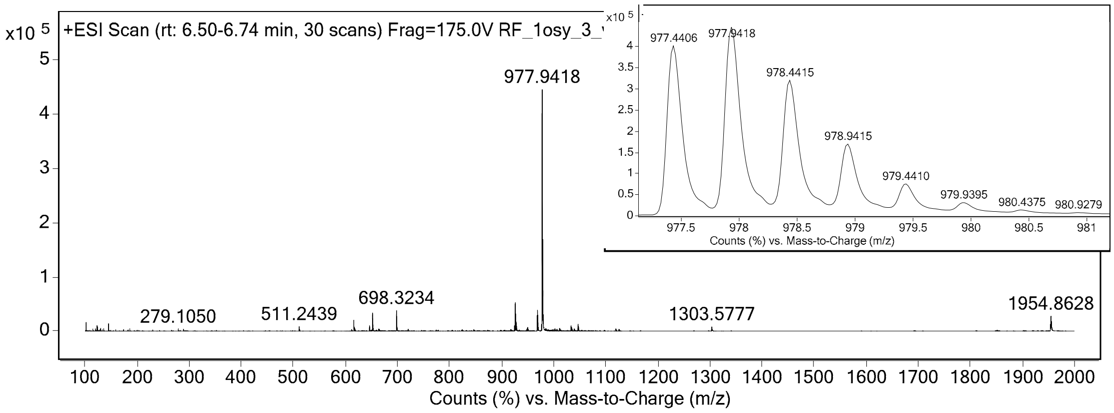

<u>**B** cyclic FAM-labeled;</u> *m/z* calculated for $C_{91}H_{114}N_{20}O_{25}S_2$; $[M+2H]^{++}$ = 976.9; found = 976.9

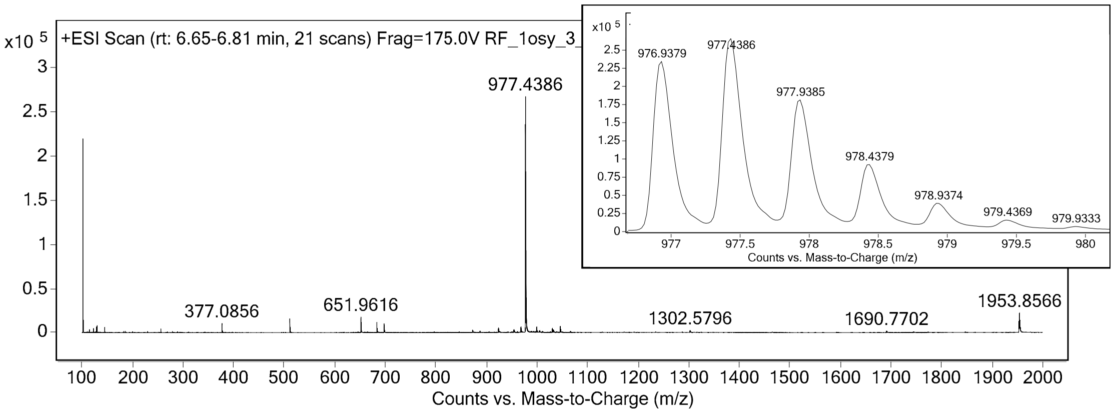

Figure S12. TIC spectra of LC-MS analysis of crude linear (A) and cyclic (B) peptide to verify cyclization by disulfie bridge formation.

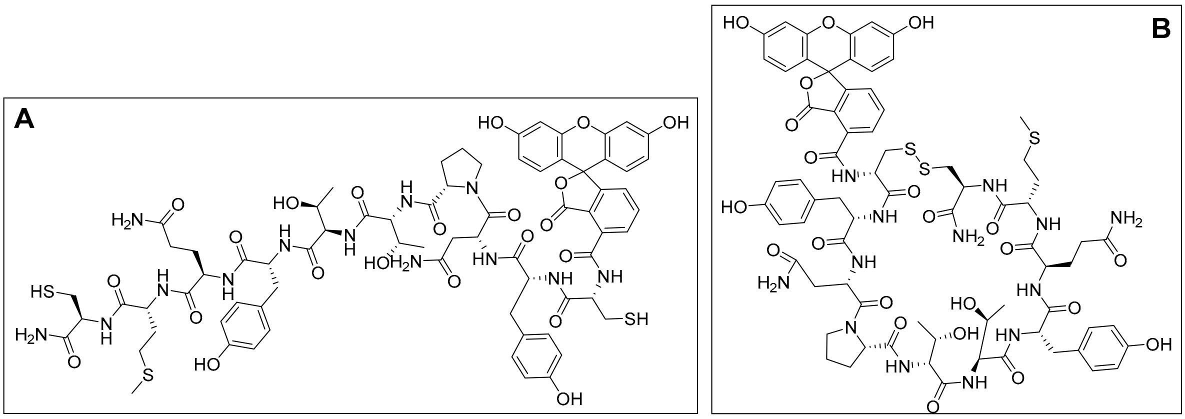

H-Cys-Tyr-Asn-Pro-Thr-Thr-Tyr-Gln-Met-Cys-NH

Figure S13. Structure and sequence information for FAM-labeled pepEDA in linear (A) and cyclic (B) variant.

<u>A linear FAM-labeled</u>; *m/z* calculated for $C_{72}H_{85}N_{13}O_{22}S_3$; $[M+2H]^{++}$ = 790.7; found = 790.7

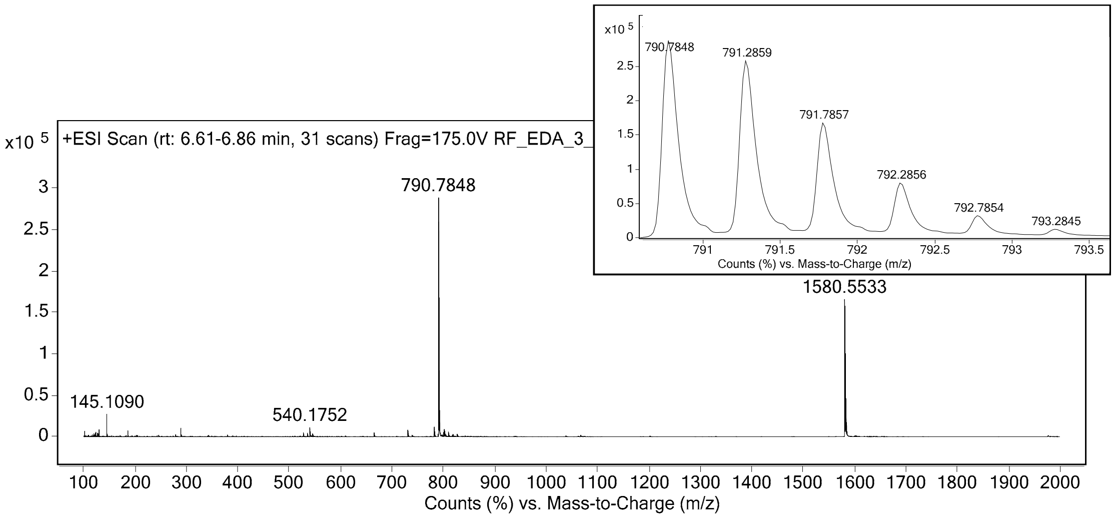

<u>B cyclic FAM-labeled</u>; *m/z* calculated for $C_{72}H_{83}N_{13}O_{22}S_3$; $[M+2H]^{++}$ = 789.7; found = 789.7

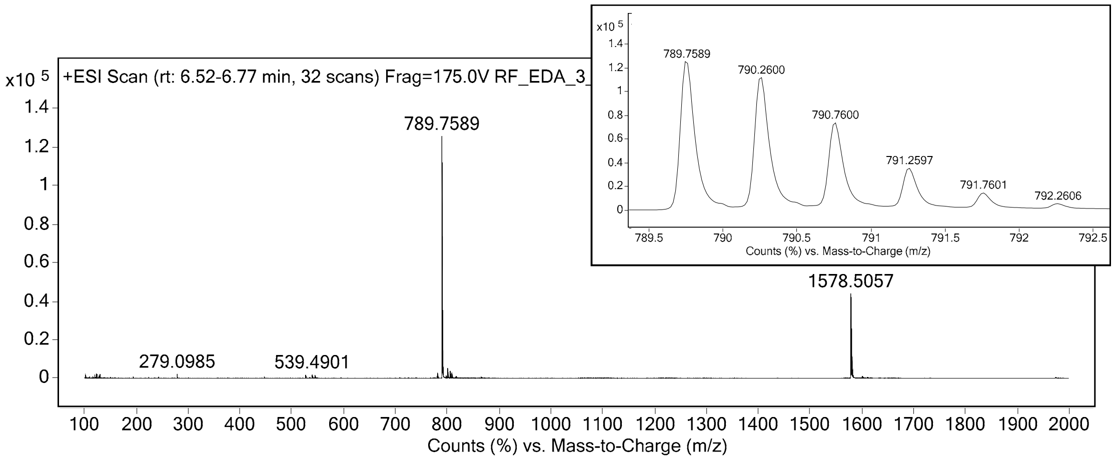

Figure S14. TIC spectra of LC-MS analysis of crude linear (A) and cyclic (B) peptide to verify cyclization by disulfie bridge formation.

3 Analytical Data of Final Compounds

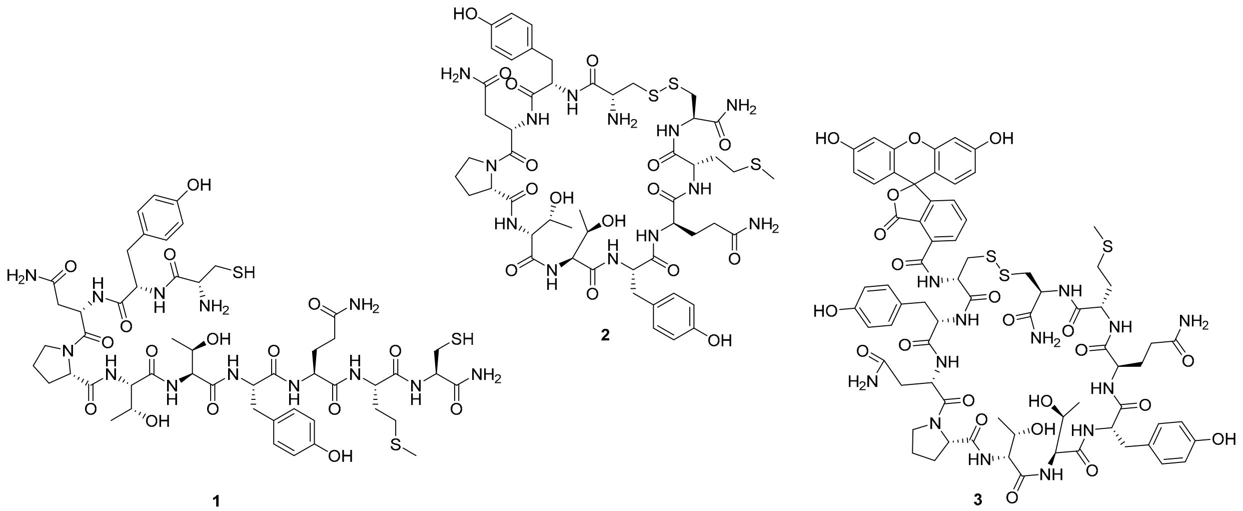

1 3

H-Cys-Tyr-Asn-Pro-Thr-Thr-Tyr-Gln-Met-Cys-NH

Figure S15. Structure and sequence information for pepEDA in linear (1), cyclic (2) and *5(6)*-carboxyfluorescein-labeled cyclic variant.

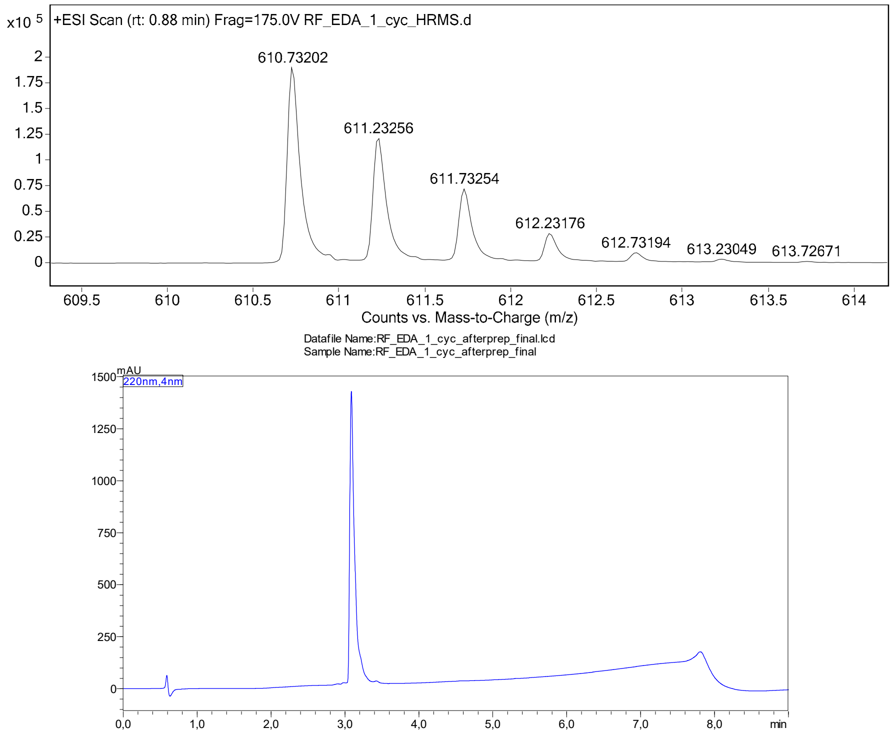

Figure S16. LC-MS and analytical HPLC data of linear (1) pepEDA.

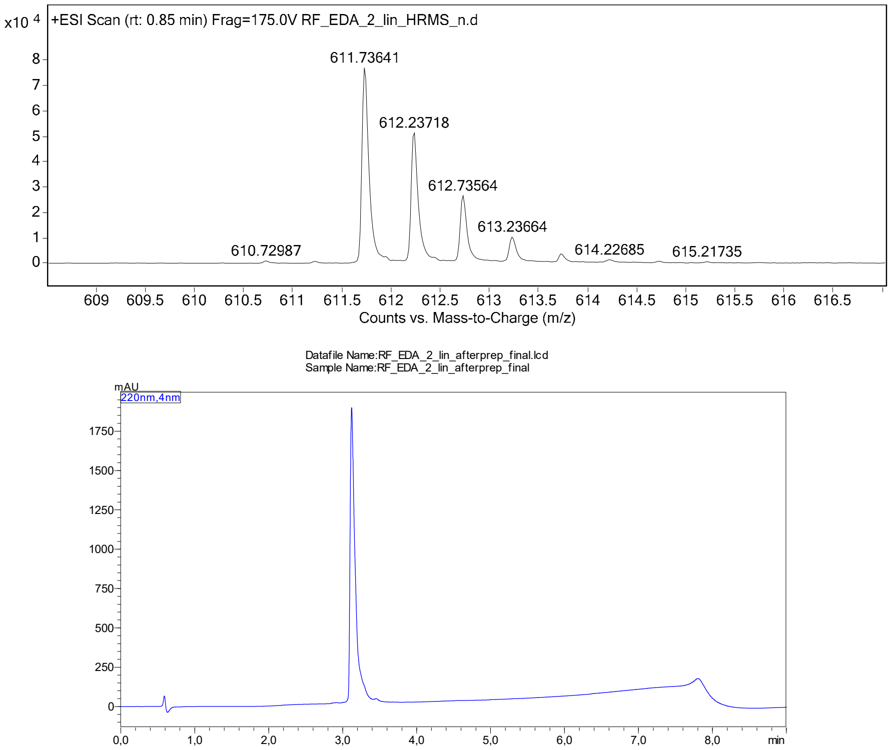

Figure S17. LC-MS and analytical HPLC data of cyclic (2) pepEDA.

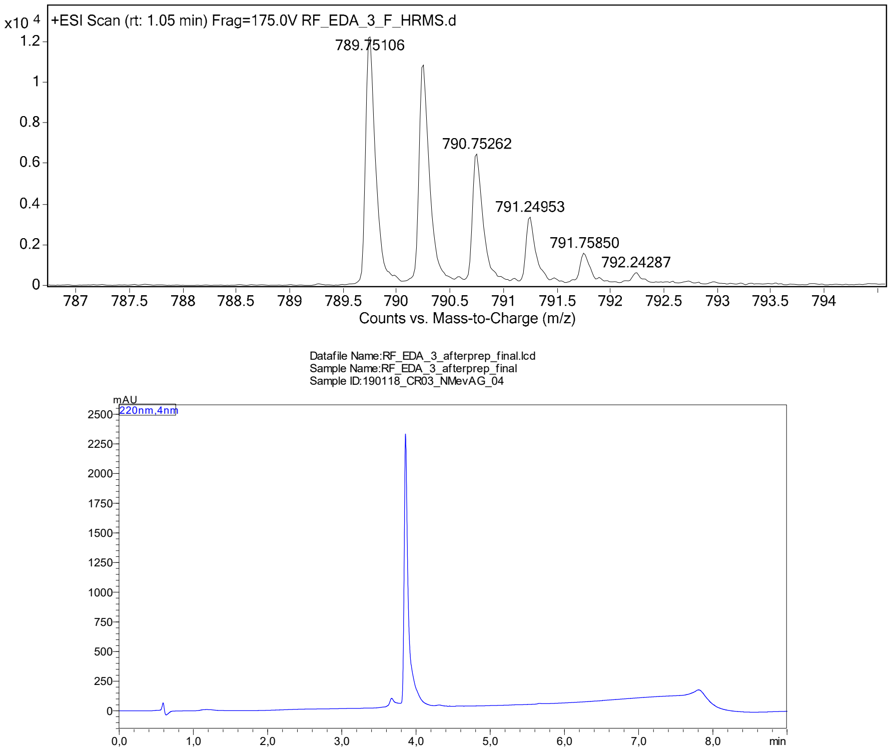

Figure S18. LC-MS and analytical HPLC data of *5(6)*-carboxyfluorescein-labeled pepEDA (3).

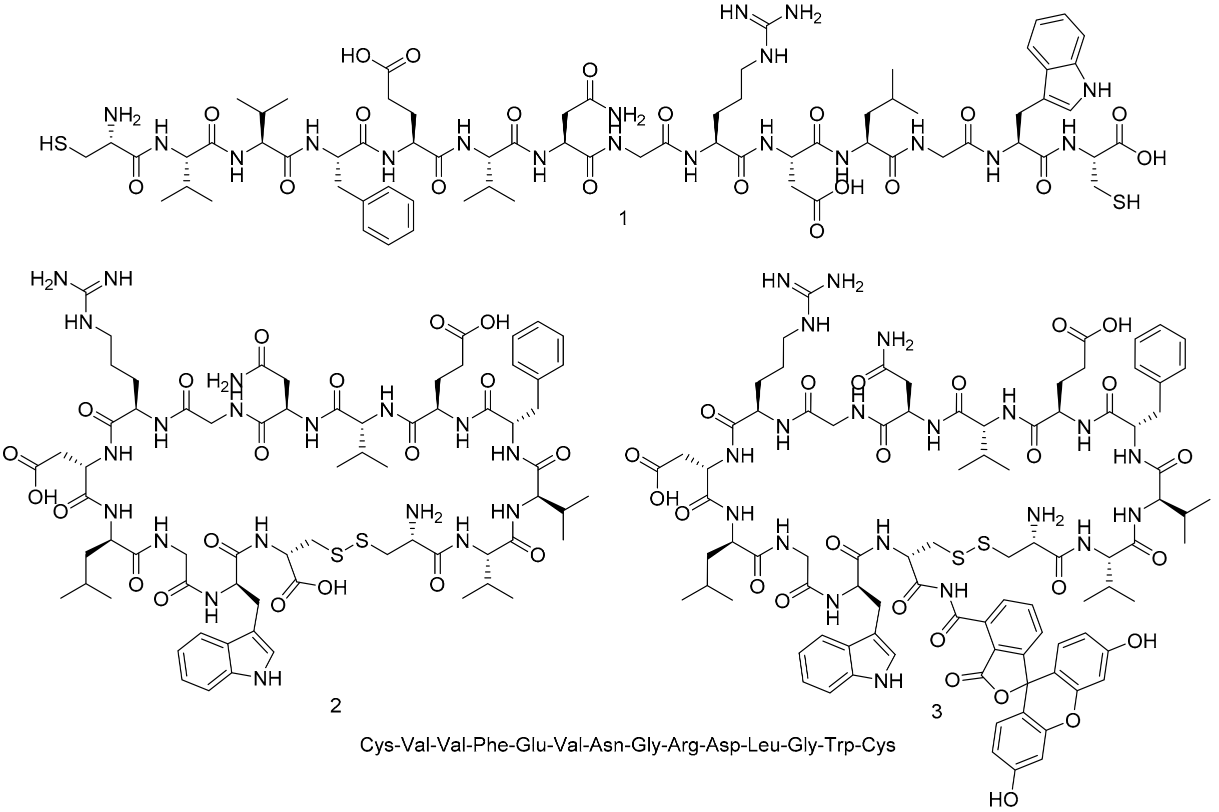

Figure S19. Structure and sequence information for pep1osy in linear (1), cyclic (2) and *5(6)*-carboxyfluorescein-labeled cyclic variant.

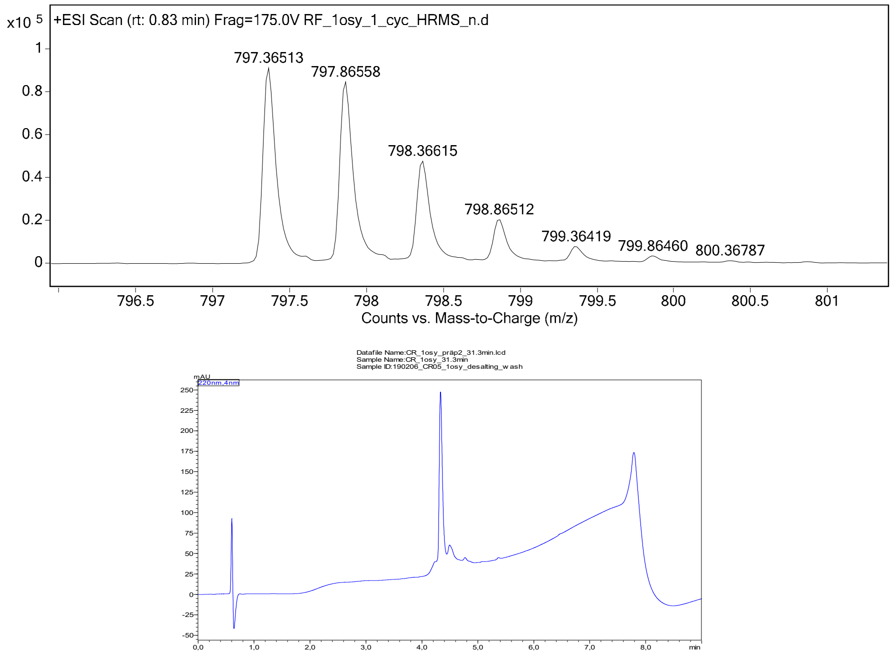

Figure S20. LC-MS and analytical HPLC data of linear (1) pep1osy.

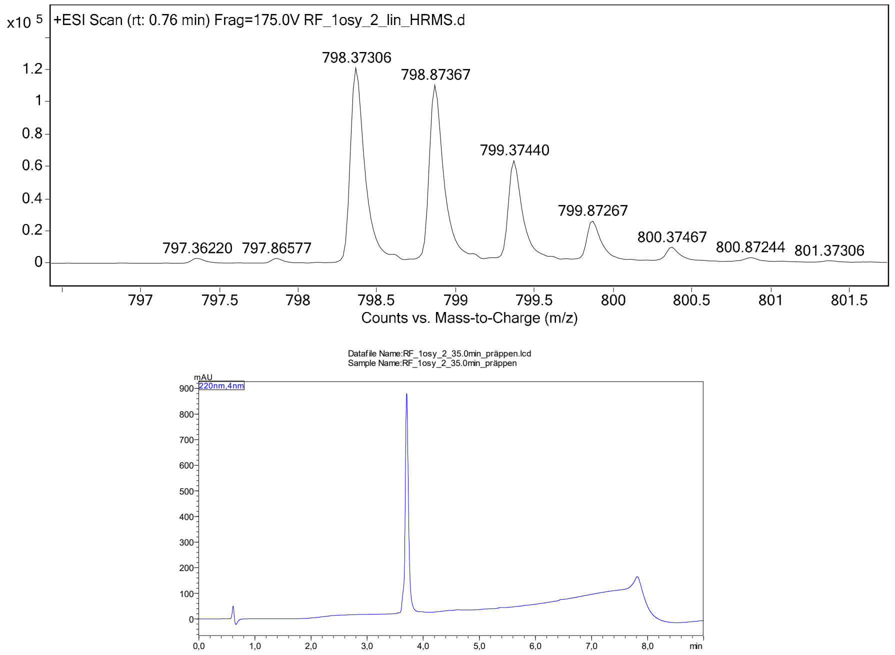

Figure S21. LC-MS and analytical HPLC data of cyclic (2) pep1osy.

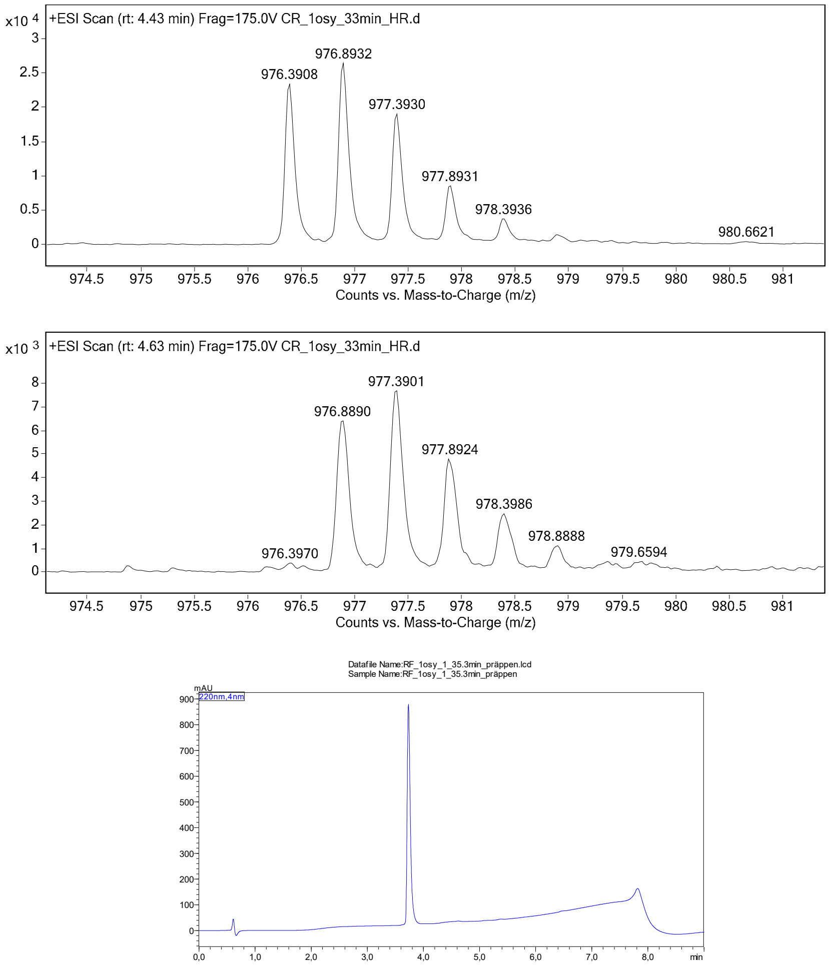

Figure S22. LC-MS and analytical HPLC data of *5(6)*-carboxyfluorescein-labeled pep1osy (3).

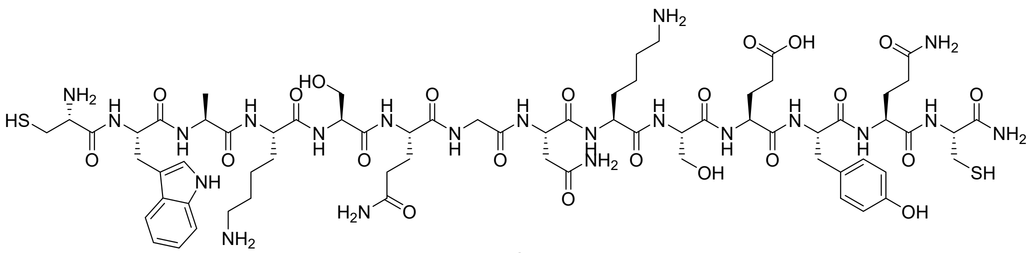

1

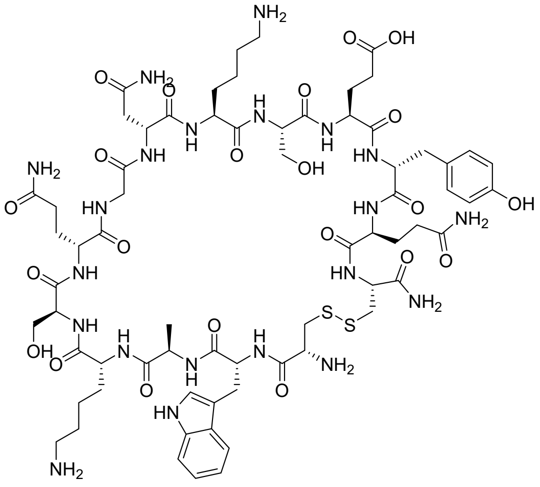

2

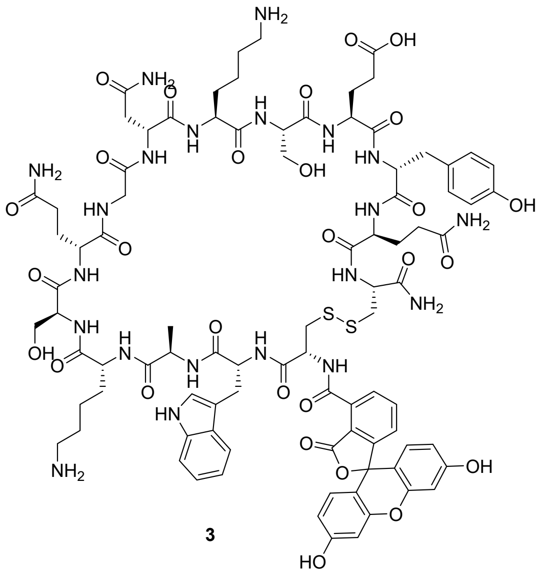

3

Cys-Trp-Ala-Lys-Ser-Gln-Gly-Asn-Lys-Ser-Glu-Tyr-Gln-Cys

Figure S23. Structure and sequence information for pep1jhf in linear (1), cyclic (2) and *5(6)*-carboxyfluorescein-labeled cyclic variant.

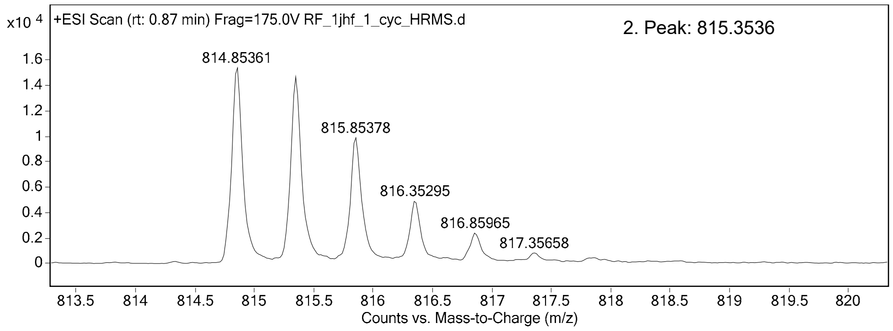

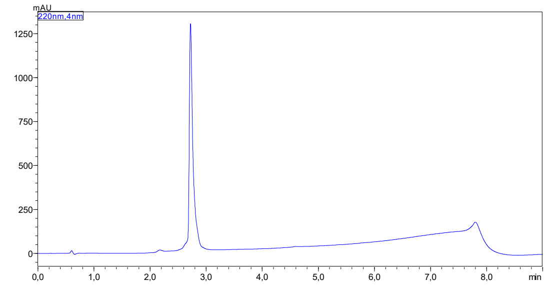

Figure S24. LC-MS and analytical HPLC data of linear (1) pep1jhf (3).

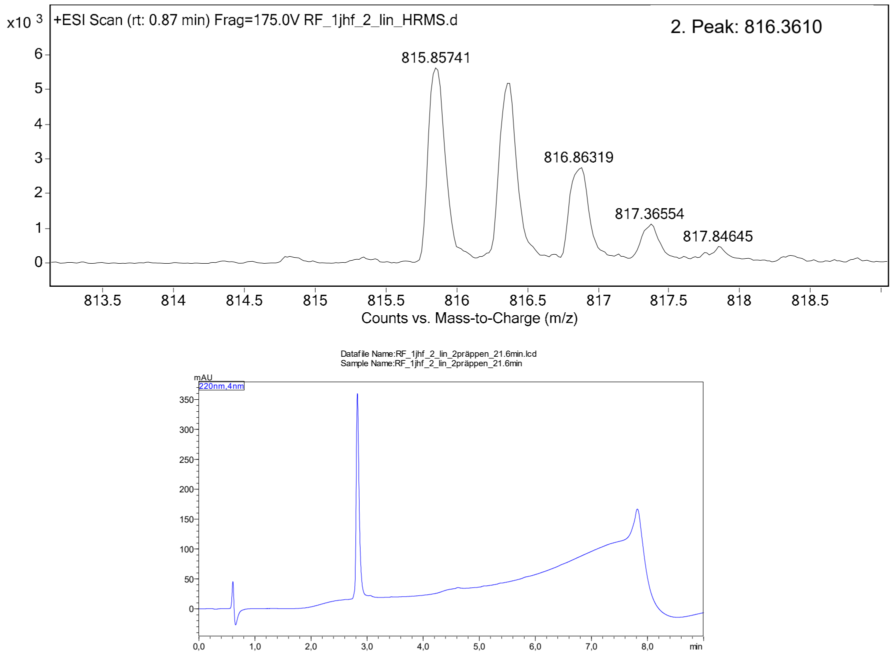

Figure S 25. LC-MS and analytical HPLC data of cyclic (2) pep1jhf (3).

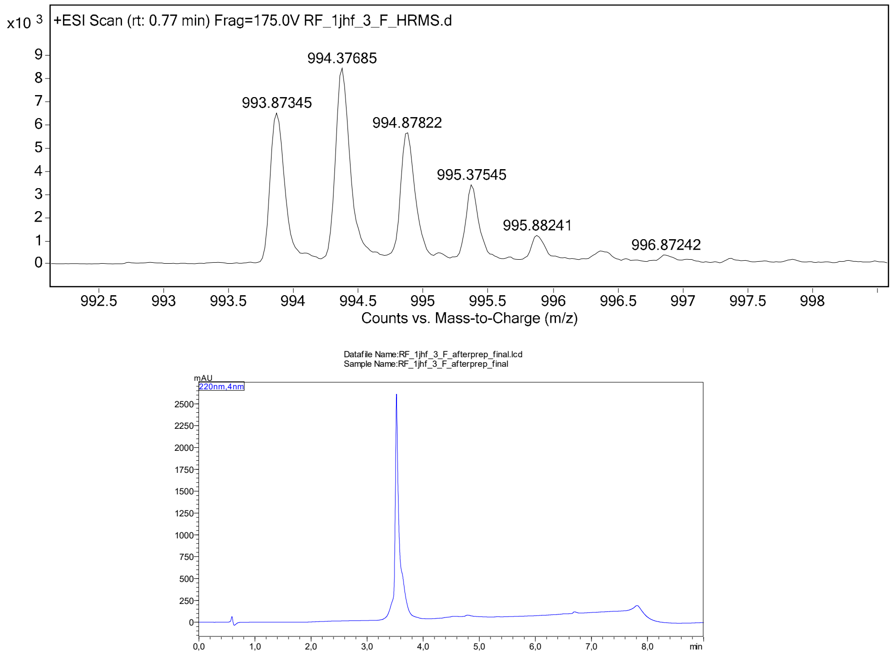

Figure S26. LC-MS and analytical HPLC data of 5(6)-carboxyfluorescein-labeled pep1jhf (3).

4 Western Blot Raw Images

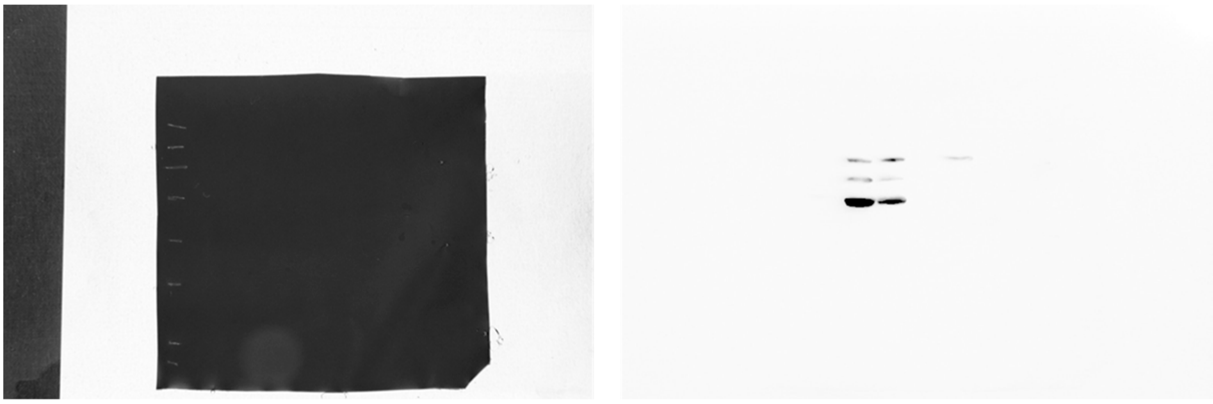

Figure S27. Uncropped, untouched, full original images of the back illuminated blot-membrane image and the bioluminescent western-blot taken with the same camera.

5 High Resolution Mass Spectrometry

	**Formula**	m/z_{calc}	m/z_{found}	**Δ ppm**
pepEDA_1	$C_{51}H_{73}N_{13}O_{16}S_3HH^{2+}$	610.7302	610.7320	2.83
pepEDA_2	$C_{51}H_{75}N_{13}O_{16}S_3HH^{2+}$	611.7381	611.7364	2.81
pepEDA_3	$C_{72}H_{83}N_{13}O_{22}S_3HH^{2+}$	789.7542	789.7510	4.01
pep1jhf_1	$C_{68}H_{101}N_{21}O_{22}S_2HH^{2+}$	814.8508	814.8536	3.39
pep1jhf_2	$C_{68}H_{103}N_{21}O_{22}S_2HH^{2+}$	815.8587	815.8574	1.56
pep1jhf_3	$C_{89}H_{111}N_{21}O_{28}S_2HH^{2+}$	993.8747	993.8734	1.33
pep1osy_1	$C_{70}H_{104}N_{20}O_{19}S_2HH^{2+}$	797.3687	797.3651	4.49
pep1osy_2	$C_{70}H_{106}N_{20}O_{19}S_2HH^{2+}$	798.3765	798.3730	4.39
pep1osy_3_Peak1	$C_{91}H_{114}N_{20}O_{25}S_2HH^{2+}$	976.3925	976.3908	1.69

1: cyclic; 2: linear, 3: cyclic + carboxyfluorescein;

6 General Analytical Methods

Gradient for preparative RP-HPLC

Flow: 10 mL/min (Hypersil Gold C_{18} column 50 × 21.2 mm, 1.9 µM particels) or 4 mL/min (Hypersil Gold C_{18} column 250 × 10.0 mm, 7 µM particels)

Eluent A: water:acetonitrile:TFA, 94.9:5:0.1; Eluent B: acetonitrile:water:TFA, 94.9:5:0.1

Eluent C: water:MeOH:TFA, 94.9:5:0.1; Eluent D: MeOH:water:TFA, 94.9:5:0.1

Method 1 for pep1osy_1, pep1osy_2, pep1jhf_1, pep1jhf_2

**min**	**Eluent A [%]**	**Eluent B [%]**
0	100	0
5	100	0
105	0	100

Method 2 for pepEDA_2

**min**	**Eluent C [%]**	**Eluent D [%]**
0	100	0
5	100	0
105	0	100

Method 3 for pep1jhf_3, pepEDA_1, pepEDA_3

min	Eluent C [%]	Eluent D [%]
0	80	20
5	80	20
85	0	100

Method 4 for pep1osy_3

min	Eluent A [%]	Eluent B [%]
0	80	20
5	80	20
85	40	60

Standard gradient for analytical RP-HPLC

Flow: 0.7 mL/min, 40 °C; Eluent A: water/TFA, 99.9:0.1; Eluent B: acetonitrile/TFA, 99.9:0.1

min	Eluent A [%]	Eluent B [%]
0	95	5
5.5	5	95
6.0	5	95
6.1	95	5
9.0	5	95

Standard gradient for LC-MS

Flow: 0.3 mL/min, 40 °C, Eluent A: water/acetonitrile/HCOOH, 94.9:5:0.1; Eluent B: acetonitrile/water/HCOOH, 94.9:5:0.1

min	Eluent A [%]	Eluent B [%]
0	98	2
10	2	98
11	2	98
11.5	100	0
15	100	0

7 Literature

1. Weller, M.L.; Amornphimoltham, P.; Schmidt, M.; Wilson, P.A.; Gutkind, J.S.; Chiorini, J.A. Epidermal growth factor receptor is a co-receptor for adeno-associated virus serotype 6. *Nat. Med.* **2010**, *16*, 662–4.
2. Sayroo, R.; Nolasco, D.; Yin, Z.; Colon-Cortes, Y.; Pandya, M.; Ling, C.; Aslanidi, G. Development of novel AAV serotype 6 based vectors with selective tropism for human cancer cells. *Gene Ther.* **2016**, *23*, 18–25.
3. Michelfelder, S.; Varadi, K.; Raupp, C.; Hunger, A.; Körbelin, J.; Pahrmann, C.; Schrepfer, S.; Müller, O.J.; Kleinschmidt, J.A.; Trepel, M.; et al. Peptide ligands incorporated into the threefold spike capsid domain to re-direct gene transduction of AAV8 and AAV9 in vivo. *PLoS One* **2011**, *6*.
